# Water-Based Highly Stretchable PEDOT:PSS/Nonionic WPU Transparent Electrode

**DOI:** 10.3390/polym14050949

**Published:** 2022-02-26

**Authors:** Youngno Kim, Sinseok Yoo, Jung-Hyun Kim

**Affiliations:** 1 KIURI Institute, Yonsei University, 50 Yonsei-ro, Seodaemoon-gu, Seoul 03722, Korea; dudsh3@yonsei.ac.kr; 2 Department of Chemical and Biomolecular Engineering, Yonsei University, 50 Yonsei-ro, Seodamoon-gu, Seoul 03722, Korea; yssangul@yonsei.ac.kr

**Keywords:** PEDOT:PSS, waterborne polyurethane, stretchable electronics, transparent electrodes

## Abstract

Poly(3,4-ethylenedioxythiophene):poly(styrenesulfonate) (PEDOT:PSS) has the merits of high electrical conductivity and solution processability, and can be dispersed in water. To improve the stretchability of PEDOT:PSS-based transparent electrode films, the intrinsically conducting polymer PEDOT:PSS was blended with highly stretchable nonionic waterborne polyurethane (WPU) and coated on a thermoplastic polyurethane (TPU) film. Nonionic WPU has good compatibility with PEDOT:PSS, without affecting the acidity. WPU undergoes hydrogen bonding and coulombic attractions with PEDOT:PSS. With variation of the WPU content, differences in the electrical properties, such as the sheet resistance and mechanical stretchability, of the coated thin films were observed. The film with 2.0 wt% WPU could be stretched to 400% of the electrode surface without damage to the surface of the electrode films. The WPU and TPU films both have a polyester group, which provides good adhesion between the WPU-based transparent electrodes and the TPU substrate films. A stretchable alternating current electroluminescence (ACEL) device was constructed by using the water-based PEDOT:PSS/nonionic WPU composite as both the bottom and top transparent electrodes. The fabricated ACEL remained its initial luminance in the 500% stretched state.

## 1. Introduction

Numerous studies have investigated stretchable electronics because of their extensive applicability to wearable electronic devices. Stretchable materials can be stretched in various forms, and are highly flexible. Therefore, stretchable materials are the most important components in the fabrication of stretchable electronic devices, such as wearable biosensors [1], wearable electronic skin [2], body motion sensors [3], and stretchable light emitting diode (LED) displays [4]. Stretchable and wearable electronic devices have recently received much attention. The intrinsically conducting polymer poly(3,4-ethylenedioxythiophene):poly(styrenesulfonate) (PEDOT:PSS) is considered an organic electrode material with excellent flexibility, which overcomes the weakness of rigid inorganic materials such as indium tin oxide (ITO) [5,6,7,8]. However, PEDOT:PSS lacks elasticity in stretchable electronics. The baking behavior of aqueous PEDOT:PSS solutions during thin film formation leads to the recrystallization of PEDOT-rich nanofibrils and chain rearrangement of both PEDOT and PSS [9,10,11]. Therefore, the baked PEDOT:PSS thin films are likely to undergo phase separation into three different regions: rigid conjugated PEDOT-rich crystalline regions, disordered PEDOT:PSS semi-crystalline regions, and PSS-rich soft regions [12]. PSS has many advantages over PEDOT complexes. However, the former is unsuitable for application in stretchable devices. This is because PSS is a relatively rigid polymer with benzene rings that form stable π–π stacks. This stable bonding structure imparts rigid properties to the conducting polymers.

Recently, several approaches have been developed to improve the stretchability of conducting polymers. Various efforts have been made to increase the elasticity by combining plasticizers with ionic additives [13,14,15]. These candidates improve the stretchability of conducting polymers and their wettability in aqueous solutions. However, the amount of additive required to enhance the stretchability is excessively high, which makes the surface of the film vulnerable to moisture and other external environmental factors. As another approach, polymerizing monomers to form conducting polymers with intrinsically stretchable structures has been attempted. Lipomi et al. synthesized a block copolymer comprising PSS and poly(poly(ethylene glycol) methyl ether acrylate (PPEGMEA) [16]. The soft and elastomeric segments, PSS-*b*-PPEGMEA, were used as a matrix to synthesize PEDOT:PSS-*b*-PPEGMEA to generate intrinsically stretchable conductive polymers. Additionally, our research group synthesized a random copolymer comprising PSS and poly(ethylene glycol) methacrylate (PEGMA), and also grafted copolymers of PSS with polyethylene glycol (PEG) to obtain stretchable conducting polymers [17,18]. Modification of PSS significantly influenced the conformational changes of the PEDOT chains and easily reduced the intrinsic electrical conductivity. The process of additional polymerization of soft copolymers to induce elasticity is complex. The most common and practical way to improve the stretchability of conducting polymers is by combination with elastomers with rubber-like properties, such as polydimethylsiloxane (PDMS) [19], polyurethane (PU) [20], and styrene-ethylene-butadiene-styrene (SEBS) [21]. These rubbery binders have superior mechanical elasticity, but disconnect the electrical pathways because of their insulating properties. Therefore, the electrical conductivity decreases sharply when the amount of elastomer exceeds the percolation threshold point [22]. It was found that as the elastomer ratio (relative to the total composition) increased, the mechanical stretchability of the electrode films improved, but the electrical conductivity decreased. Specific optimization of the electrical conductivity to achieve balance with the stretchability is required to prevent the properties from being skewed to one side in a trade-off relationship.

In this study, nonionic waterborne polyurethane (WPU), which is an elastomer that is dispersible in water, is selected to enhance the stretchability of PEDOT:PSS. The advantages of using WPU include high deformability, non-flammability, transparency, and water resistance after film formation. Notably, WPU is highly compatible with PEDOT:PSS and both can be combined through a simple blending process. The nonionic properties of WPU are maintained over a wide pH range, and the dispersion stability is not affected by acid or alkali [23]. Additionally, functional groups such as –NH_2_ and the O atoms of WPU undergo strong interactions with the –SO_3_H groups of PSS. Therefore, WPU and PEDOT:PSS are compatible with each other, without phase separation of the blends [24]. The effect of the WPU ratio on the electrical conductivity and deformability of the PEDOT:PSS/nonionic WPU composite is evaluated. The PEDOT:PSS/nonionic WPU solution with the optimal composition is coated on thermoplastic polyurethane (TPU) substrate films. This assembly is suitable for wearable electronics because of the eco-friendly and harmless properties of the components [25,26,27].

## 2. Materials and Methods

### 2.1. Materials

**Figure 1:**
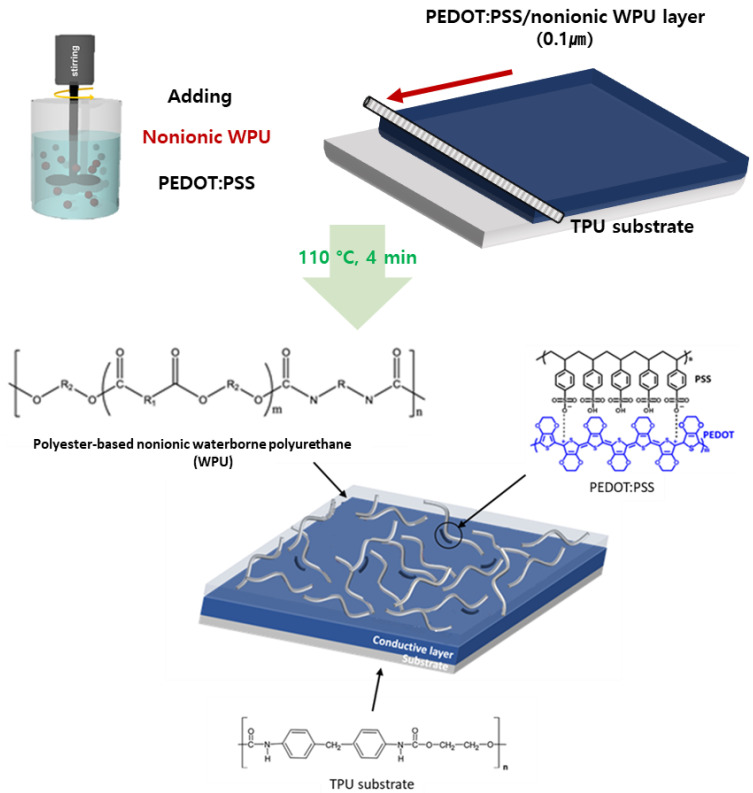
Fabrication Process and Structure of Stretchable Electrode Film. (In WPU, R, R1, and R2 indicate different alkyl or aryl groups).

An aqueous PEDOT:PSS dispersion was synthesized using the Baytron P procedure [28]. The mass ratio of PEDOT to PSS was 1:2.5 wt%. The solid PEDOT:PSS solution was diluted to 1.0 wt%. The pH of the PEDOT:PSS solution was adjusted from 1.5 to 7 using sulfuric acid and ammonia. Ethylene glycol (EG 98%) was purchased from Sigma-Aldrich Co., Yongin-si, Gyeonggi-do, Korea. Waterborne polyurethane (WPU-3501D), a polyester-based nonionic polyurethane aqueous dispersion, was purchased from Taiwan PU Corporation (Figure 1; R, R1, and R2 represent different alkyl or aryl groups) at a concentration of 39.8 wt%. The thermoplastic elastomers used in this study, including the TPU film (thickness = 200 µm; Woojinpackage, Seoul, Korea) were commercially available. ZnS/Cu phosphor microparticles were purchased from Shanghai Keyan Phosphor Technology Company, Shanghai, China. Silicone rubber (Ecoflex 00-30) was purchased from Smooth-on. Macungie, PA, USA.

### 2.2. Preparation of Stretchable Electrodes

#### Fabrication of Stretchable Electrode Films 

First, EG 7.0 wt% and fluorosurfactant (FS-31; 0.1 wt%) were added to the PEDOT:PSS aqueous dispersions, stirred for 10 min, and filtered through a 0.45 µm polypropylene syringe filter. WPU was added to the PEDOT:PSS dispersion at different concentrations. The PEDOT:PSS/nonionic WPU dispersion was coated on a TPU substrate using an RDS (RD Specialties, Inc., Webster, NY, USA) coating bar and baked in a convection oven at 110 °C for 4 min. The sheet resistance of the PEDOT:PSS thin film was determined by the ratio of WPU. The thickness of transparent electrode film with transmittance was shown in Table S1.

### 2.3. Preparation of Alternative Current Electroluminescence (ACEL) Device 

The ACEL devices were fabricated using PEDOT:PSS/nonionic WPU on TPU films as both electrodes, with the following device structure: TPU Film|PEDOT:PSS/nonionic WPU electrode|ZnS:Cu embedded in silicone rubber|PEDOT:PSS/nonionic WPU electrode|TPU Film. ZnS:Cu embedded in silicone rubber was prepared by mixing in a weight ratio of 1:2 (ZnS:Cu/silicone rubber). The ZnS:Cu/silicone rubber composite was spin-coated onto the PEDOT:PSS/nonionic WPU electrode film at 400 rpm for 30 s and then laminated onto the other stretchable electrode film. The ACEL device film was then baked at 85 °C for 30 min. The size of the device was 10 cm × 2 cm, the emission area was 2 cm × 2 cm, and the device was stretched using a custom stretching machine. The devices was then fabricated and operated at room temperature.

### 2.4. Sample Preparation and Characterization

The samples were prepared using the procedure described above; the thickness of the films was approximately 100 nm. Morphological analysis of the PEDOT:PSS/nonionic WPU films was conducted using a field emission scanning electron microscope (FE-SEM, JEOL-7800F, JEOL Ltd., Tokyo, Japan). The morphology of the thin films was observed from the topography and phase images acquired using atomic force microscopy (AFM; XE-100, Park Systems, Suwon-si, Korea). Fourier transform infrared (FTIR) spectra were obtained using an FTIR spectrometer in attenuated total reflection (ATR) mode (model Vertex 70, Bruker, Billerica, MA, USA). The sheet resistance was measured using the 4-point probe method (RT-70V, Napson, Tokyo, Japan); a schematic image is shown in Figure S1. When measuring the sheet resistance, the voltage condition was always fixed to 10 mV automatically. The film thickness was measured using the surface profiler (DektakXT Stylus Pro-filer, Bruker). The electrical conductivity of the stretchable electrode film was calculated using Equation (1).

Electrical Conductivity (σ) [S/cm] = 1/Resistivity (ρ) [ohm·cm] = 1/Sheet resistance [ohm/sq] × Thickness [cm](1)

The luminance of the ACEL device film was measured using a spectrophotometer (CS-2000, Minolta, Osaka, Japan) with an AC power supply (APS-7050, GWInstek, Xinbei, Taiwan) under ambient air.

## 3. Results

### 3.1. PEDOT:PSS/Nonionic WPU Composite

#### 3.1.1. Compatibility of PEDOT:PSS and Nonionic WPU

**Figure 2:**
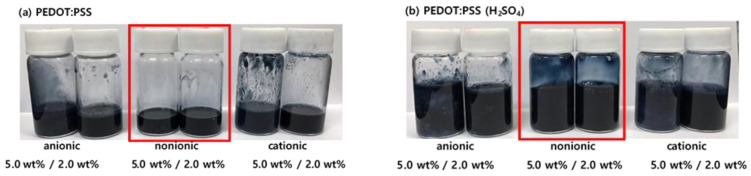
(**a**) Compatibility of PEDOT:PSS and (**b**) sulfuric acid-doped PEDOT:PSS with different types of WPU.

Basically, the PEDOT chains are attached with PSS anions by electrostatic attraction and PSS roles the primary dopants for PEDOT. In this study, the chemical doping level was controlled through additional doping with H_2_SO_4_ or neutralization with ammonia. Sulfuric acid induced the PEDOT chains to form a crystalline nanofibril structure, in which positively charged PEDOT and negatively charged PSS were segregated [29]. As explained in more detail in Section 3.1.2, the intrinsic electrical conductivity of PEDOT:PSS influenced the maintenance of sheet resistance at stretched state. Cationic and anionic WPU undergo aggregation due to pH-induced collision. Figure 2a shows the aggregation of both positive and negative ions. Nonionic WPU has good compatibility with PEDOT:PSS over a wide pH range, as well as good dispersion properties, and storage stability. Figure 2b shows the effect of the R–group of WPU on the compatibility with PEDOT:PSS doped with sulfuric acid [23,30].

Anionic sulfone-containing groups, phosphonates, and carboxylic acids are the most commonly used R–groups. Polyurethane contains cationic groups, tertiary sulfur atoms, or quaternary nitrogen atoms. These ionic groups induce aggregation because the dispersion balance in the emulsion is broken owing to the reaction of the PSS acid.

**Figure 3:**
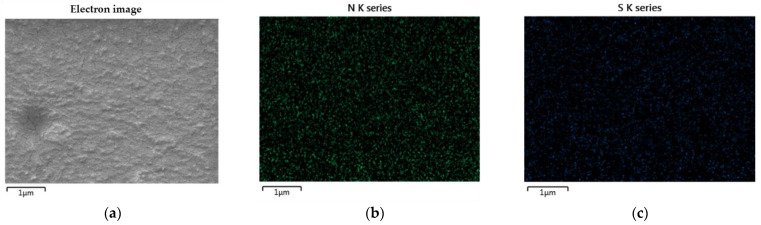
Scanning electron microscope (SEM) images and EDS elemental mapping images: (**a**) electron image; (**b**) nitrogen and (**c**) sulfur distribution in PEDOT:PSS/nonionic WPU (WPU 2.0 wt%) on TPU film.

This characteristic indicates that PEDOT:PSS can be mixed with WPU. The excellent compatibility of PEDOT:PSS with nonionic WPU was further demonstrated by EDS. Figure 3 shows the nitrogen and sulfur distribution in the PEDOT:PSS/nonionic WPU composite with 2.0 wt% WPU, coated on the TPU film. The nitrogen atoms come from the WPU and TPU films, while the sulfur atoms come from PEDOT:PSS. Nitrogen and sulfur were evenly distributed throughout the coating layer. These results indicate that the PEDOT:PSS/nonionic WPU forms a miscible polymer blend. The good miscibility of these two polymers can be attributed to their strong interactions. Hydrogen bonds may form between PSS and WPU because of the oxygen atoms in PSS and the NH groups in WPU. In addition, protons can be transferred from the –SO_3_H of PSS to the –NH of WPU. As a result, –SO_3_H is converted to negatively charged –SO_3_^−^, whereas –NH is converted to positively charged –NH_2_^+^. Therefore, there is a coulombic attraction between PSS and WPU [31].

**Figure 4:**
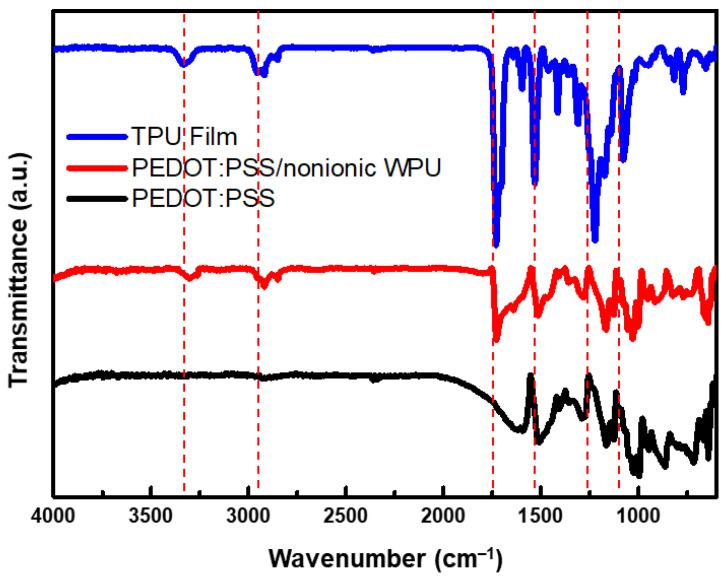
FTIR spectra of TPU film (Bare), PEDOT:PSS/nonionic WPU, and PEDOT:PSS coated on TPU film.

As shown in Figure 4, The peaks between 3320 and 3335 cm^−1^ in the FTIR spectra of the films are attributed to the urethane and urea N–H groups. Two peaks located at approximately 3320 and 3450 cm^−1^ are often observed, which can be assigned to hydrogen bonding and non-hydrogen bonding N–H of the urethane and urea groups [32]. In this case, only a single peak at approximately 3333 cm^−1^ was visible, suggesting that most of the N–H groups are involved in hydrogen bonding. In this region, oscillatory peaks that gain intensity with the nature of the C=O group and its hydrogen bonding capacity appeared at different wavenumbers in the profile of WPU. Sharp peaks were distinguished at approximately 1720 cm^−1^, and shoulders were observed at approximately 1700 cm^−1^, assigned to the C=O groups of free urethane and the polyester groups, and hydrogen-bonded C=O of the urethane groups, respectively [32]. 

Nonionic WPU has good compatibility with acids. It exhibits good dispersibility and adhesion to the PSSH segments without PEDOT attached. It forms hydrogen bonds and coulombic interactions with PEDOT:PSS [33,34]. Acid doping is required to improve the electrical conductivity of PEDOT:PSS, and sufficient acid doping can be achieved in nonionic WPUs.

#### 3.1.2. Preparation and Surface Morphology of the PEDOT:PSS/Nonionic WPU

To understand the role of WPU in improving the mechanical properties of PEDOT:PSS, AFM was used to investigate the surface morphology of the samples with and without WPU. The effects of the sulfuric acid doping level on the morphology of the films was observed by AFM imaging. The morphology of sulfuric-acid-treated PEDOT:PSS was uniform (Figure S2a). The AFM image of the film with 2.0 wt% of WPU added to PEDOT:PSS solution shows that WPU was well dispersed, giving rise to a uniform topology (Figure S2d,e).

**Figure 5:**
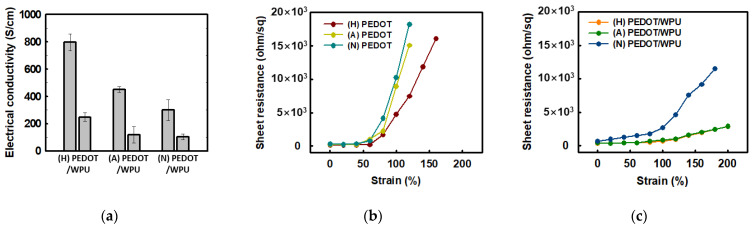
(**a**) Electrical conductivity of PEDOT:PSS (left) and PEDOT:PSS/nonionic WPU (2.0 wt%) (right) with PEDOT:PSS under different pH conditions; (**b**) sheet resistance of PEDOT:PSS films when TPU substrates were strained from 0% to 200%. (**c**) Sheet resistance of PEDOT:PSS films with PEDO:PSS/WPU (2.0 wt% WPU) when TPU substrates were strained from 0% to 200%. (N = PEDOT:PSS (neutral), A = PEDOT:PSS (acid), H = PEDOT:PSS (doped with sulfuric acid)).

The effect of the pH of PEDOT:PSS type on the electrical conductivity of the PEDOT:PSS solution with 2.0 wt% WPU is shown in Figure 5. For the PEDOT:PSS sulfuric acid-doped, acid(non-treated), and neutralized samples, the electrical conductivity changed from 799.2 to 248.3 S/cm, 450.0 to 117.3 S/cm, and 300.0 to 102.8 S/cm respectively. This is because WPU plays a non-conductive role in PEDOT:PSS, resulting in lower electrical conductivity. Although the same amount of WPU was added to each, the PEDOT:PSS doped with sulfuric acid/nonionic WPU showed the highest electrical conductivity. Even after the addition of the WPU, the electrical conductivity tended to be the same as the initial value for each PEDOT:PSS with different pH as shown in Figure 5a. In the composite with WPU, the soft WPU segment improved the adhesion to the TPU substrate film and the role of the matrix, resulting in less increase in the sheet resistance, even up to 200% strain (see Figure 5c). When nonionic WPU was used, better electrical conductivity could be obtained even at high strain, due to the compatibility with the sulfuric acid-doped PEDOT:PSS.

#### 3.1.3. Improvement in Mechanical Properties of PEDOT:PSS/Nonionic WPU

**Figure 6:**
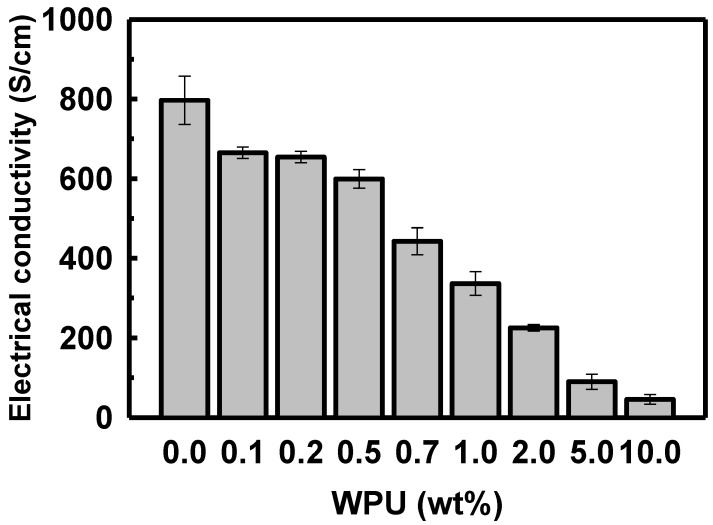
Electrical conductivity of PEDOT:PSS/nonionic WPU with various proportions of WPU in the PEDOT:PSS solution.

When the WPU loading in the solution of sulfuric acid-doped PEDOT:PSS was increased to 0.1, 0.2, 0.5, 0.7, 1.0, 2.0, 5.0, and 10.0 wt%, the electrical conductivity decreased to 655.0, 654.1, 599.2, 442.6, 336.6, 225.3, 89.8, and 45.4 S/cm, respectively (see Figure 6). The electrical conductivity of the polymer blend decreased sharply when the WPU content exceeded 2.0 wt%.

**Figure 7:**
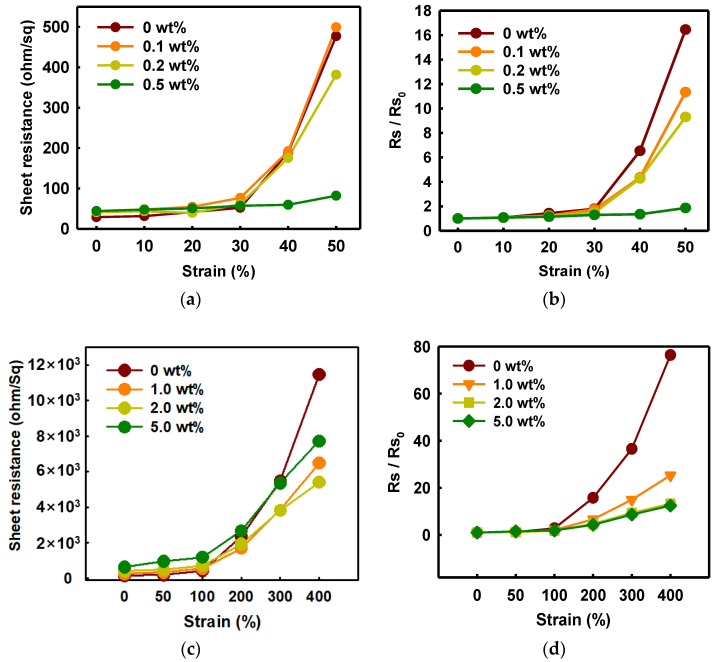

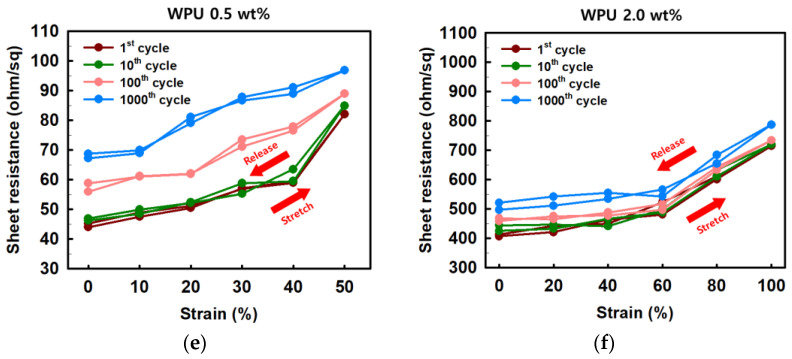
(**a**) Sheet resistance and (**b**) normalized sheet resistance of PEDOT:PSS films with various concentrations of WPU when TPU substrates were strained from 0% to 50%. (**c**) Sheet resistance and (**d**) normalized sheet resistance of PEDOT:PSS films with various concentrations of WPU when TPU substrates were strained from 0% to 400%. Strain–sheet resistance curves of PEDOT:PSS/nonionic WPU under stretch–release: (**e**) WPU 0.5 wt% at 50% strain and (**f**) WPU 2.0 wt% at 100% strain.

As shown in Figure 7, the change of the sheet resistance of PEDOT:PSS/nonionic WPU was observed for specific WPU contents divided into a range of low and high elongation. When the WPU content became more than 0.5 wt%, the sheet resistance was almost maintained even at 50% strain. Since only a little WPU was added, the initial sheet resistance was superior at 44 ohm/sq (Figure 7a,b). However, the initial sheet resistance increased a lot, approximately 400 ohm/sq, and the initial value maintained well even at 100% strain when the WPU content increased to 2.0 wt% (Figure 7c,d).

**Figure 8:**
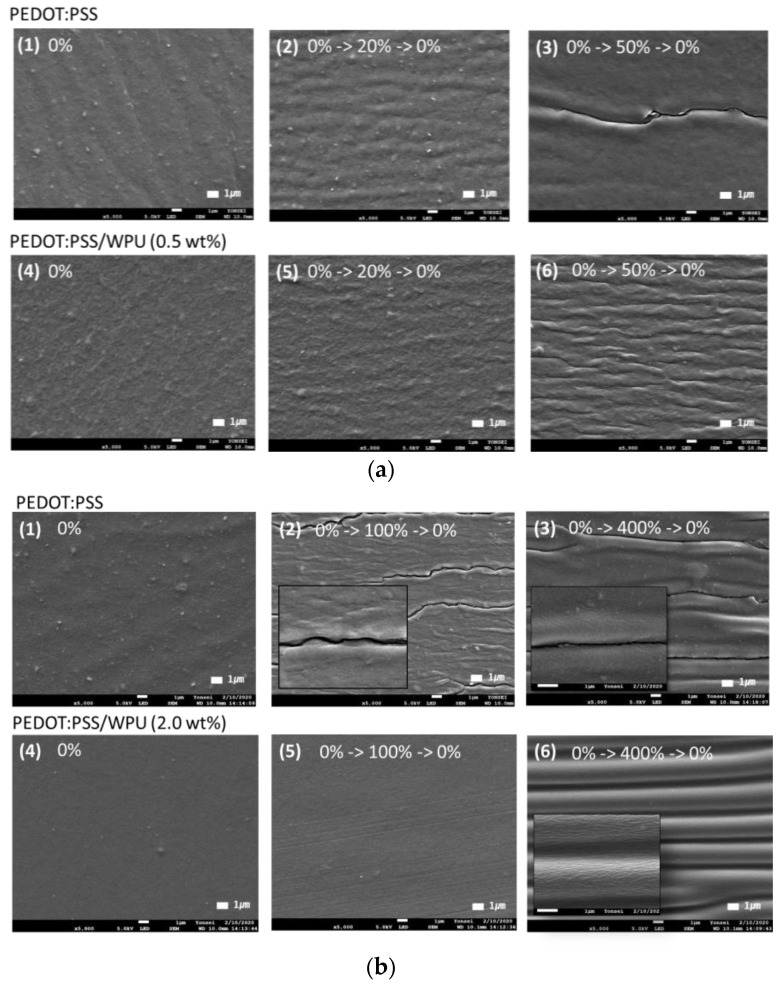
Scanning electron microscope (SEM) images of (**a**) PEDOT:PSS only and PEDOT:PSS/nonionic WPU (0.5 wt%) coated on TPU substrate after strain and release with 20 and 50% strain. (**b**) PEDOT:PSS only and PEDOT:PSS/nonionic WPU (2.0 wt%) coated on TPU substrate after strain and release with 50 and 100% strain. (**1**) and (**4**) under 0% strain, (**2**), (**3**), (**5**) and (**6**) after one stretch-release different strains.

Through morphology analysis before and after stretching, the crack that interferes with the electrical pathway was observed. In the case of 50% strain or higher, the only PEDOT:PSS film showed the crack, whereas the PEDOT:PSS/nonionic WPU 0.5 wt% showed the wrinkled surface that dissipated the fore under elongation (see Figure 8(a3,a6)). The morphology images matched the change of sheet resistance of Figure 7. The WPU content required for 50% strain and 100% strain was optimized to 0.5 wt% and 2.0 wt%, respectively.

As shown in Figure 7e,f, the stretch–release cycling test was conducted by the PEDOT:PSS/nonionic WPU 0.5 wt% at strain 50% and PEDOT:PSS/nonionic WPU 2.0 wt% at strain 100%. In both cases, the stretchable electrode films showed the superior deformability that presented the recovering with the initial sheet resistance after stretch–release. Even after 1000 cycle repetitions of stretch–release, the sheet resistance only changed by 1.5 times.

#### 3.1.4. Mechanical Properties of Other Substrate Materials

**Figure 9:**
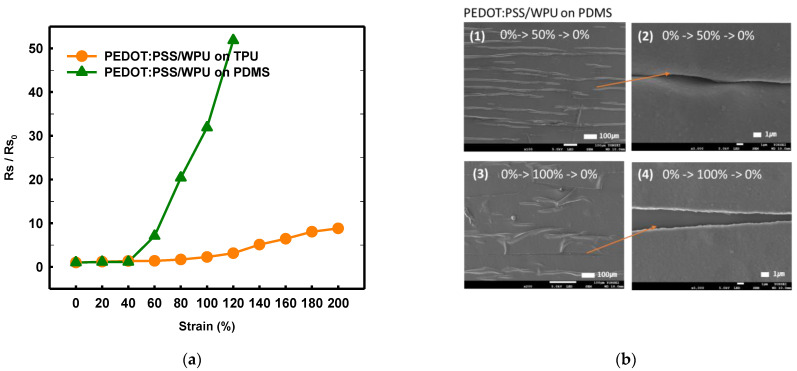
(**a**) Normalized sheet resistance of PEDOT:PSS/nonionic WPU (2.0 wt%) on TPU film and PEDOT:PSS/nonionic WPU on PDMS under strain from 0% to 200%. (**b**) Scanning electron microscope images of PEDOT:PSS/nonionic WPU (2.0 wt%) on TPU film and PEDOT:PSS/nonionic WPU on PDMS after strain and release with 50 and 100% strain.

By comparing the TPU and PDMS substrates, it was possible to observe the change in the adhesion and sheet resistance according to the strain that emerges owing to the difference in the substrate (see Figure 9a). Both the WPU and TPU films have reactive polyester groups; therefore, the adhesion was good. With the PDMS substrate, the sheet resistance increased sharply above 50%, even when WPU was added. Because the PDMS surface is hydrophobic, the strain and coating layers peeled off when subjected to different strains (Figure 9b). the TPU with high surface energy as the substrate film improved the deformability of PEDOT:PSS/nonionic WPU more than the PDMS with low surface energy.

### 3.2. Alternating Current Electroluminescent (ACEL) Device

Stretchable ACEL Device Employing PEDOT:PSS/Nonionic WPU Composite Electrodes on TPU Film

**Figure 10:**
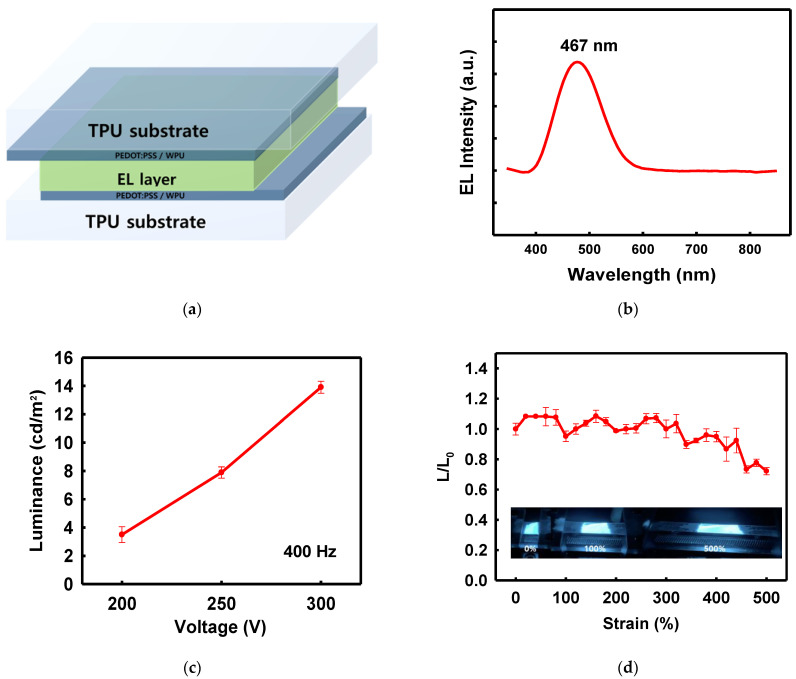
(**a**) Schematic of device structure, (**b**) electroluminescence spectrum of the device, centered at 467 nm, (**c**) luminescence according to voltage change at 400 Hz, (**d**) *L/L*_0_ ratio of the device under strain and photographs of the device under 0, 100, and 500% strain.

The PEDOT:PSS/nonionic WPU composite electrodes on the TPU film were used in a demonstration ACEL device. A ZnS:Cu phosphor mixed with silicone rubber acted as a stretchable light-emitting layer between the two electrodes. Figure 10a shows the structure of the ACEL device manufactured using the solution process. The ACEL device exhibited excellent elastic motion without degradation of the performance, even at 500% strain (Figure 10d). The ACEL device was powered by a rectangular pulse function (pulse voltage of 300 V and frequency of 400 Hz) and showed a maximum luminance of 14.29 cd/m^2^ from the emission peak at 467 nm, at a voltage of 300 V (Figure 10b,c).

Figure 10d shows the deformation-dependent properties of the stretchable ACEL devices. The stretchable ACEL device maintained the 70% of the initial luminance at 500% stretched state, showing very stable emission performance (Figure 10d).

## 4. Conclusions

A facile approach for improving the stretchability of electrode films by mixing highly conductive PEDOT:PSS with a highly stretchable nonionic WPU was presented. The PEDOT:PSS solution could be blended with nonionic WPU over a wide range of PEDOT:PSS ratios. The intrinsically conducting polymer PEDOT:PSS was miscible and dispersed within the WPU matrix of the composite films. In addition, WPU showed good bonding strength with PSS and acted as an excellent bonding agent for the TPU substrates. The initial electrical conductivity of the composite films decreased as the WPU weight percentage increased, whereas the elasticity was improved. The intrinsic electrical conductivity of PEDOT:PSS was strongly influenced by the WPU content within specific compositional ranges. The WPU content required for 50% strain and 100% strain was optimized to 0.5 wt% and 2.0 wt%, respectively. The stretchable ACEL device employing the PEDOT:PSS/nonionic WPU electrode films could be stretched up to 500% strain. The stretchable and transparent electrode films are applicable in fully stretchable ACELs.

## Data Availability

All data produced in this study are available on request from the corresponding author.

## Supplementary Materials

The following supporting information can be downloaded at: https://www.mdpi.com/article/10.3390/polym14050949/s1, Table S1: Coating thickness and transmittance of PEDOT:PSS/nonionic WPU film.; Figure S1: The schematic image of sheet resistance measurement by 4-point probe system (Napson / RT-70V); Figure S2: AFM 2D topographic (left), phase (middle) and 3D topographic (right) images of the PEDOT:PSS coated on TPU films, (a) PEDOT:PSS doped with sulfuric acid, (b) PEDOT:PSS and (c) neutralized PEDOT:PSS with ammonia solution. (d) PEDOT:PSS doped with sulfuric acid/nonionic WPU, (e) PEDOT:PSS/nonionic WPU, and (f) neutralized PEDOT:PSS with ammonia/nonionic WPU. All images are measured by 5 × 5 μm^2^.
