# Supplementary material for: Water-Based Highly Stretchable PEDOT:PSS/Nonionic WPU Transparent Electrode
Source: Polymers (Basel). 2022 Feb 26;14(5):949. doi: 10.3390/polym14050949 (PMC8912668; doi:10.3390/polym14050949)
Supplement: Supplementary file 1 [file polymers-14-00949-s001.zip › polymers-1617777-supplementary.pdf]

Supplementary Information

Water-based Highly Stretchable PEDOT:PSS/nonionic WPU Transparent Electrode

Youngno Kim [1,†], Sinseok Yoo [2,†] and Jung-Hyun Kim [2,*]

[1] KIURI Institute, Yonsei University, 50 Yonsei-ro, Seodaemoon-gu, Seoul 03722, South Korea; dudsh3@naver.com
[2] Department of Chemical and Biomolecular Engineering, Yonsei University, 50 Yonsei-ro, Seodamoon-gu, Seoul 03722, South Korea; yssangul@yonsei.ac.kr
* Correspondence: jayhkim@yonsei.ac.kr
† These authors contributed equally to this work.

Table S1. Coating thickness and transmittance of PEDOT:PSS/nonionic WPU film.

Sample	Thickness (μm)	Transmittance (%)
TPU Film (bare)	200	90.0
PEDOT:PSS (#15 bar)	0.34	80.0
PEDOT:PSS/WPU (#15 bar)	0.34	77.0
PEDOT:PSS (#40 bar)	0.91	67.0
PEDOT:PSS/WPU (#40 bar)	0.91	68.0

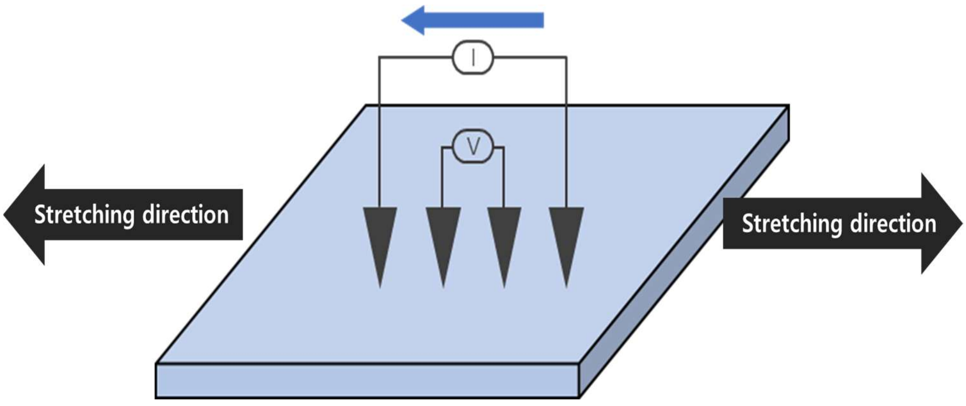

Figure S1. The schematic image of sheet resistance measurement by 4-point probe system (Napson / RT-70V).

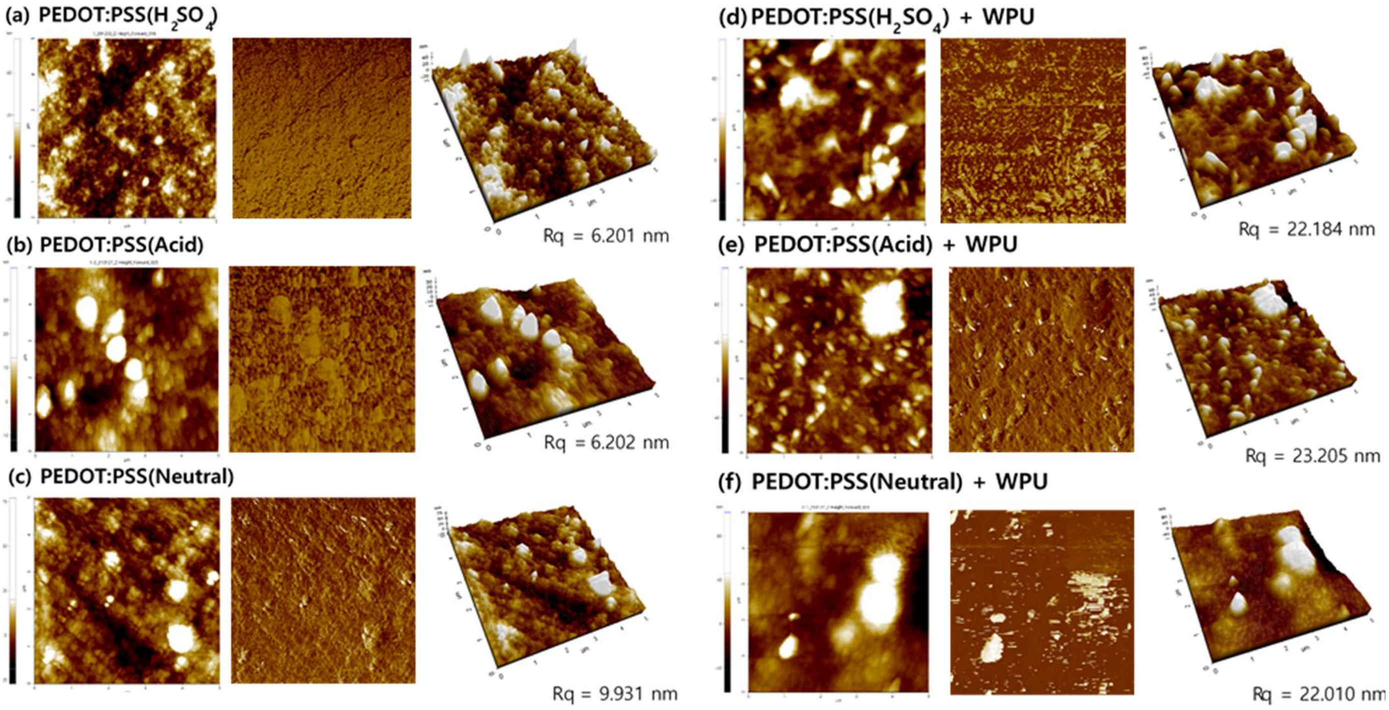

Figure S2. AFM 2D topographic (left), phase (middle) and 3D topographic (right) images of the PEDOT:PSS coated on TPU films, (a) PEDOT:PSS doped with sulfuric acid, (b) PEDOT:PSS and (c) neutralized PEDOT:PSS with ammonia solution. (d) PEDOT:PSS doped with sulfuric acid/nonionic WPU, (e) PEDOT:PSS/nonionic WPU, and (f) neutralized PEDOT:PSS with ammonia/nonionic WPU. All images are measured by 5 × 5 μm^2.
